# Change in sediment transport regime of the Keelung River in Taiwan induced by the operation of Yuanshantze flood diversion tunnel

**DOI:** 10.1371/journal.pone.0311551

**Published:** 2024-12-26

**Authors:** Tsung-Yu Lee, Tse-Yang Teng, Jun-Yi Lee, Yen-Wei Pan, Ming Chen, Chi-Cheng Chiu

**Affiliations:** 1 Department of Geography, National Taiwan Normal University, Taipei, Taiwan; 2 Department of Natural Resources and Environmental Management, University of Hawaiʻi at Mānoa, Honolulu, Hawaii, United States of America; Chang’an University, CHINA

## Abstract

The impact of flood diversion channels on river sediment transport has been rarely reported. This study uses the Yuanshantze flood diversion tunnel (YFDT), which was commissioned in July 2005 in Taiwan, as an example. This study calculates the sediment transport in the Keelung River from 1997 to 2018 by using seasonal rating curves, in the form of *aQ*^*b*^. Changes in rating curve coefficients are also analyzed to understand the impact of YFDT on sediment transport regime. The results show that after the construction of YFDT, the annual sediment transport dropped from 0.59 ± 0.47 [Mt y^-1^] to 0.17 ± 0.09 [Mt y^-1^], leading to dampened inter- and intra-annual variation. Before flood diversion, the Keelung River requires ~1% cumulative time to export 50% cumulated sediment loads, but it takes ~4.5% cumulative time after flood diversion. Exponent *b* decreased from 1.23±0.18 to 1.15±0.13, and *log a* decreased from 0.71±0.15 to 0.51±0.11, suggesting that the Keelung River is akin to a different river in terms of sediment transport regime. While the design of the diversion tunnel mainly considered its impact on flow, its impact on sediment transport is far greater than its impact on flow and should not be overlooked. Whether this new normality will affect the downstream river continuum requires continuous attention.

## Introduction

The amount of sediment from land to the ocean has always been a focal point of interest [1, 2]. Fluvial sediment export is tightly related to the evolution of geomorphology [3, 4], changes in coastlines [5, 6], off-shore aquatic ecosystems [7, 8], physical and chemical weathering [9, 10]. Considering the carbon cycle, rivers connect the atmosphere to the ocean, carrying organic carbon in soil from the contemporary carbon fixed in plants to be buried deep in the ocean, functioning as a global warming counteracting mechanism [11–13]. Anthropogenic influences and changing climate have affected the supply and flux of sediment along hydrological pathways [14]. Infrastructures, e.g., water diversion schemes and dams, decelerate the sediment flux to the coastal zone [15–17]. However, previous studies on the influence of hydraulic structures in rivers on sediment transport have mostly focused on the impact of dams or reservoirs [18, 19], with limited research on the impact of flood diversion channels on fluvial sediment transport.

Various flood control measures are employed in rivers to reduce flood risks, such as flood bypasses, reconnected floodplains, compound channels, backwaters, distributaries, and inter-basin transfers [20]. However, distributaries or distributary channels, which are streams that branch off and flow away from a main stream channel to another basin or directly to the sea, are uncommon. Currently, there are few engineered distributaries that discharge into the sea worldwide, such as the Mississippi River in the United States, the Sarawak River in Malaysia, and the Turia River in Spain. These three diversion channels are all located within deltas. To reduce the risk of flooding in the Taipei metropolitan area, Taiwan, the Yuanshantze flood diversion tunnel (YFDT) was constructed in the upstream of the Keelung River, one of the three main tributaries feeding into the Taipei metropolitan area. This has effectively increased the safety of downstream residents [21]. However, the YFDT in Taiwan is unique in that it was excavated closed to the headwater, diverting some of the floodwaters directly into the East China Sea. The Keelung River, where the YFDT is located, stretches about 93 kilometers from its origin to the sea outlet, with the diversion tunnel about 70 kilometers from the sea outlet. The length of the river section affected by the diversion, as a percentage of the total river length, is likely the largest among all similar diversion projects.

Taiwan is a subtropical mountainous island with a maximum elevation of approximately 4,000 meters above sea level and about 70% of its area located above 100 meters above sea level. The island-wide annual rainfall is around 2,400 mm, which is over three times the global average [22]. Due to its steep slopes and small watersheds, about 70% of the annual rainfall in Taiwan turns into runoff. From May to October, Taiwan is affected by 3–5 typhoons, which bring torrential rainfall with high intensity, resulting in episodic floods. Additionally, intense rainfall, combined with high tectonic rates, leads to rapid mass wasting and fluvial sediment transfer, resulting in Taiwan having the most turbid rivers and the highest sediment yields among the world’s rivers [4, 23–26]. Previous studies have shown that rainfall extremes in Taiwan have increased by approximately 90% [27], leading to an increase of over 100% in runoff extremes [28] and even more significant increases in sediment transport [13]. The design of the YFDT only considers its impact on flow, and its effect on sediment transport may also be worth investigating.

The rating curve method is one of the most appropriate load estimation methods and has been widely applied to rivers in Taiwan, particularly suitable for the sediment load estimation [13, 29–31]. Kao et al. (2005) has applied seasonal rating curves in the form of power function, i.e. *y = ax*^*b*^, with bias correction factors to the 16 Taiwan rivers [29]. Kao and Milliman (2008) has utilized the estimates of the daily sediment load to explain the role of lithology, episodic events, and human activities on sediment load from Taiwan rivers [31]. Lee et al. (2015) has demonstrated good capability of this procedure for Taiwan rivers, further revealing the mean daily sediment export from Taiwan Island in the recent stage (1990–2010) significantly increased by >80% (compared to values in 1970–1990) with subtle increase in daily runoff [13]. However, the data from the Keelung River were not included in their analysis.

Moreover, sediment transport regime can be discussed via the time-variant coefficients of the rating curves, i.e. *a* and *b* in the power function [32]. If the coefficients of the rating curves (regardless of whether the curves are established at monthly or annual time scales) are plotted with *log a* on the x-axis and exponent *b* on the y-axis, all points will fall on a negatively correlated straight line [33]. This straight line is considered a good indication of the sediment transport regime for a river, with all gauges that plot on the same line characterized by a similar sediment transport regime [33]. The sediment transport regime is typically consistent even if at different positions along the same river [34, 35]. However, Sun et al. (2020) used the *b-log a* pairs and found the changes of sediment transport regime after the large-scale soil and water conservation measures (called ecological restoration) in the Middle Yellow River Basin [36]. However, whether it is soil and water conservation or reservoir construction, sediment is retained within the watershed, unlike the YFDT, which directly diverts sediment away from the watershed. How this affects the sediment transport regime of the Keelung River also warrants investigation.

To understand the impact of the construction of YFDT on sediment load in the Keelung River, this study focuses on the Wudu discharge gauge located downstream of YFDT. Using sediment concentration and water discharge data observed at the gauge station, the rating curve method is employed to estimate the daily sediment load through the Wudu gauge station and to quantify the differences before and after the construction of the YFDT. Additionally, by analyzing the changes in rating curve coefficients over time, this study investigates whether the sediment transport regime has changed before and after the construction of the YFDT. The specific steps carried out in this study are as follows: (1) Collect continuous discharge data and discrete suspended sediment concentration data monitored by the Water Resources Agency (WRA) at the Wudu gauge station from 1997 to 2018. (2) Using the discharge and sediment concentration data, establish seasonal rating curves and calculate the bias correction factors. (3) Input the continuous discharge data into the rating curves with bias correction factors derived in the previous step to calculate the daily sediment load passing through the Wudu gauge station. (4) Use the start of the YFDT operation in July 2005 as a boundary to divide the data into pre-flood diversion (Pre-FD) and post-flood diversion (Post-FD) periods, and analyze the changes in sediment load and sediment transport regime. The findings of this study will serve as a reference for the overall sustainable management of the watershed.

## Material and methods

### Yuanshantze flood diversion tunnel (YFDT)

**Fig 1:**
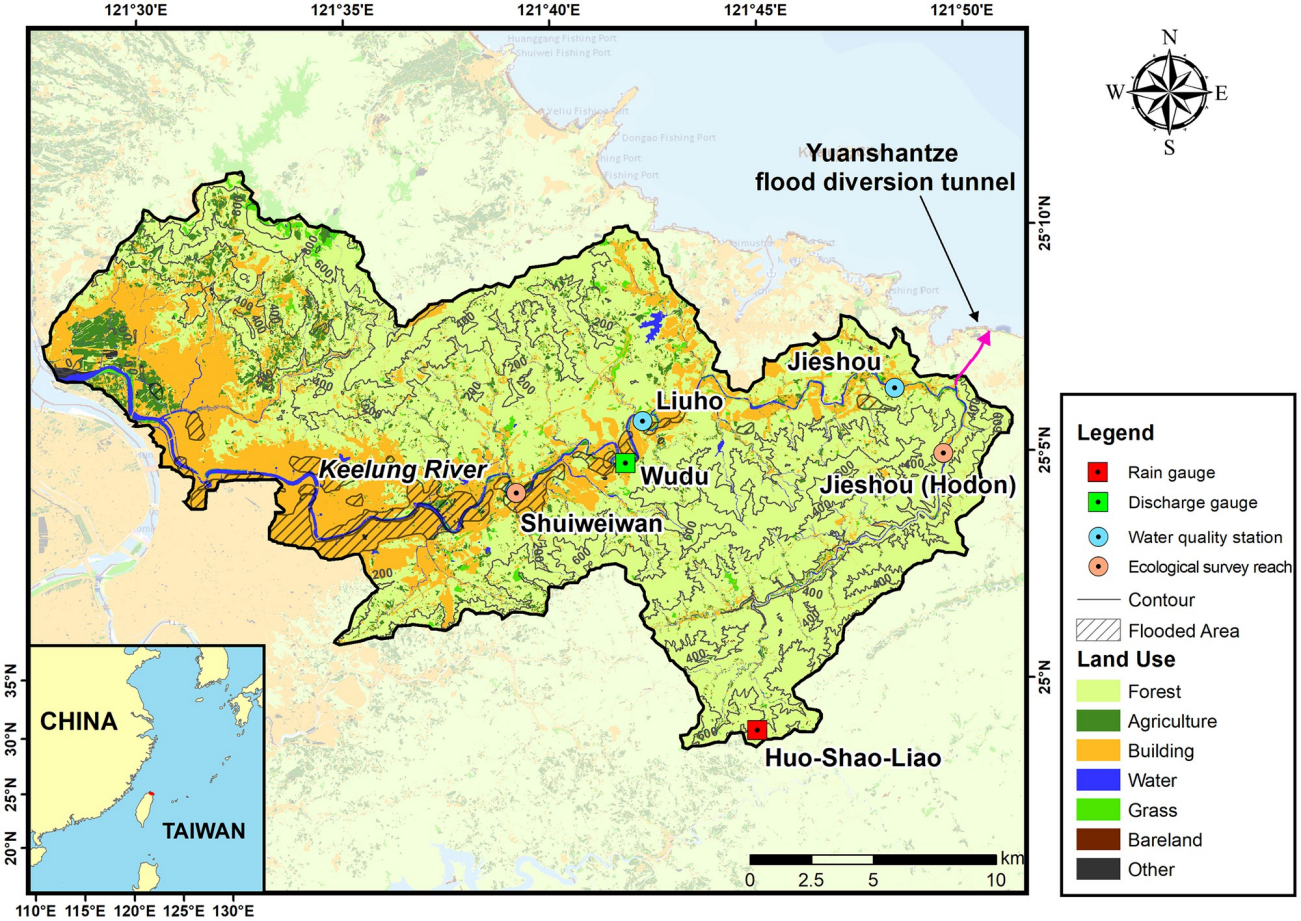
The map of land use and location of Yuanshantze flood diversion tunnel in the Keelung River watershed. The land use data, published in 2005, are sourced from the Ministry of Interior. The location of Wudu discharge gauge and Huo-Shao-Liao rain gauge are marked. Jieshuo and Liuho are water quality stations, and Jieshuo(Hodon) and Shuiweiwan are two ecological survey reaches. The shaded area represents the flooded area caused by Typhoon Nari in 2001.

The Keelung River is one of the three major tributaries flowing through the Taipei metropolitan area, with a watershed area of 491 km^2^ and a tributary length of 86 km. The terrain along both sides of the river is characterized by a river valley, and the river meanders with a gentle slope. Due to its proximity to the Taipei metropolitan area, extensive development has taken place on both banks, leading to severe conflicts between waterway path and human activities. Additionally, influenced by its geographical location and topography, the mountainous areas in the watershed serve as a center for rainfall, making the region prone to heavy rain during typhoons, resulting in flash floods and mountain torrents. Consequently, low-lying areas often suffer from flood disasters. In the highly developed areas along both banks, where the river channel is constrained, flooding occurs frequently, particularly in the middle and lower reaches. In particular, the damage caused by Typhoon Nari in 2001 was the most severe, resulting in a total of 6,640 ha of flooding in the Taipei metropolitan area. The flooded area in the Keelung River basin alone was approximately 2,281 ha, as indicated by the shaded area in Fig 1.

In response to this, the WRA implemented the YFDT project, which commenced in 2002 and was completed in early 2005. This project involved the construction of an 8-meter-high, 30-meter-long check dam in the upper reaches of the Keelung River. A side weir was also installed on the upstream right side of the check dam. When the water level reaches 63 meters, the flood is naturally diverted through an overflow weir into a 2,483-meter-long flood diversion tunnel with a diameter of 12 meters, directing the floodwater directly into the East China Sea. After the completion of this project, during the arrival of a 200-year frequency flood with a water discharge of 1,620 [m^3^sec^-1^] in the upper reaches, 1,310 [m^3^sec^-1^] can be diverted through the tunnel into the East China Sea. The remaining flow of 310 [m^3^sec^-1^] enters the middle and lower reaches, serving ecological and water quality purification purposes. The flood diversion result can effectively reduce the flood level by an average of 1.5 meters in the middle and lower reaches, demonstrating significant flood mitigation effects. Furthermore, it helps avoid the acquisition of a large amount of private land and the improvement of bridges in the middle and lower reaches, reducing the overall cost of the Keelung River management project, and promoting local prosperity and ensuring the safety of people’s lives and properties [21, 37].

### Hydrometric data

**Fig 2:**
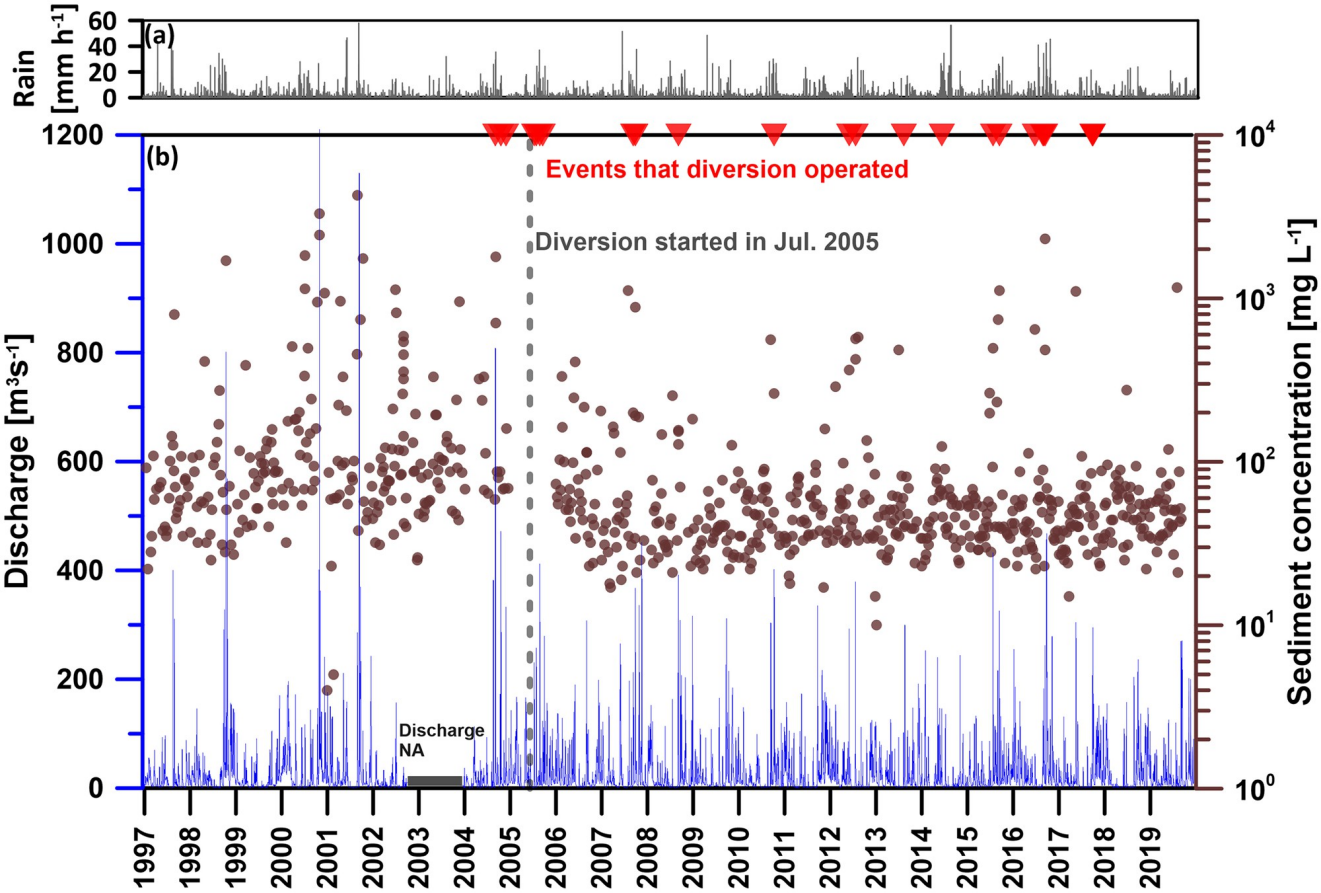
(a) The rainfall data measured at the Huo-Shao-Liao rain gauge and (b) the discharge and sediment concentration data measured at the Wudu gauge station. The hourly rainfall are in grey. The daily discharge are in blue and the biweekly sediment concentration are in brown. The YFDT was officially commissioned in July 2005. Prior to its official operation, it had been utilized three times for emergency flood diversion. All periods during which the YFDT was functional are marked with red triangular symbols.

The hydrometric data of were provided by the WRA of Taiwan. At Wudu discharge gauge, water discharge (*Q*) was measured daily (or hourly for the typhoon / rainstorm events) and the suspended sediment concentration (*Cs*) was measured biweekly by using USDH-48 depth-integrated suspended sediment sampler recommended by the Federal Interagency Sedimentation Project of the USA [4]. The drainage area of the Wudu gauge station is 204 [km^2^], located approximately 21 [km] downstream from the YFDT (Fig 1). It is the closest gauge station to the YFDT, and its records of discharge and sediment concentration have been documented since 1997, encompassing data before and after the YFDT construction. This comprehensive dataset can be utilized to assess the impact of YFDT on river discharge and sediment transport. The discharge and sediment concentration data measured at the Wudu gauge station are shown in Fig 2. The YFDT was officially commissioned in July 2005. Prior to its official operation, it had been utilized three times for emergency flood diversion. For the subsequent analysis, July 2005 will be used as a reference point to understand the influence of YFDT on both river discharge and sediment transport. Until 2018, the YFDT has executed a total of 24 flood diversion missions. Among these, there were 2 occurrences in June and July each, 5 occurrences in August, 7 occurrences in both September and October, and 1 occurrence in December. Out of the 24 missions, only 7 were triggered by rainstorm, while the rest were a result of typhoon invasions. Besides, the hourly rainfall data from the Huo-Shao-Liao rain gauge, maintained by Central Weather Administration, are used to reveal the possible effects of rainfall pattern changes on the changes of water discharge and sediment.

### Sediment load estimation

Calculating sediment discharge of a river is straightforward if *Q* [m^3^s^-1^]and *Cs* [mg L^-1^] are measured continuously at closely spaced intervals. However, in most cases continuous records of *Cs* are usually not available, so indirect methods such as sediment rating curves, as in this study, must be utilized. Kao et al. (2005) developed stratified time-frame rating curves in the form of power function (i.e. *Qs = Q‧Cs‧0*.*086 = aQ*^*b*^ where *Qs* indicates sediment load [t d^-1^]) [29]. To develop meaningful rating curves for each time frame, they determined an optimal procedure to separate yearly data into low-flow (November-May) and high-flow (June-October) months and used a FORTRAN program that incorporates limited-range extrapolation in water discharge, representative data points, and meaningful regressions. The coefficient *a* and exponent *b* in the power function for each seasonal rating curve can be derived from the observed sediment loads and the river discharge by the log-linear least-square method, i.e. *log Qs = log a + b log Q*. The separate rating curves in a year were then applied to daily (or hourly for the events) discharges to calculate daily suspended sediment load. However, sediment loads obtained by log-linear regression following a back log-transformation are well known to have underestimated predictions. To overcome the bias, several statistical techniques have been developed such as the maximum likelihood estimate [38, 39], minimum variance unbiased estimate [40, 41], non-parametric smearing estimator [39, 42], and stratified rating curves [43, 44]. Overall, these statistical techniques are devised to overcome underestimation by conventional rating curve method. However, in practical applications, the aforementioned correction method presents two issues: it either still underestimates the total observed sediment load or further overestimates the already overestimated estimates. In contrast to the previous bias-corrections, which are based on residuals in log-transformed units and back-transformed again before using them. Kao et al. (2005) define residuals in non-log transformed units (*ε*_*i*_ = *Qs*_*i*_*-aQ*_*i*_^*b*^, where *ε* is the residual error between observation and estimation and *i* is every individual sediment sample) in their bias-factor calculation [29]. The bias-correction factor, *β*, for each rating curve, is defined as the sum of residuals divided by the sum of total estimations. The equation for the corrected estimation is given by: *(1+β) ‧aQ*^*b*^. Since *Qs* in Taiwan expands over 6 orders of magnitude, the larger *Qs* dominates the total sediment load more during the studied period [13]. Much larger residual errors always appear at high-flow estimations, which have heavier weighting in determining *β* value while being compared to those residuals for low-flow estimations. Accordingly, this *β* factor can efficiently modify the rating curve toward the largest *Qs*, which means the largest sediment load in a respective dataset could be estimated better. Meanwhile, positive and negative residuals in the corrected equation will be balanced out and the sum of total estimations will approximate the sum of total observations. When combined with hourly discharge data, the method successfully estimated *Qs* in response to episodic events, particularly for the measurements at the higher-end which are most important in sediment export estimation. Estimation error is thus significantly reduced. For a detailed explanation of the methods, please refer to Kao et al. (2005) [29].

## Results

### Effects of YFDT on variation of discharge and sediment concentration

**Fig 3:**
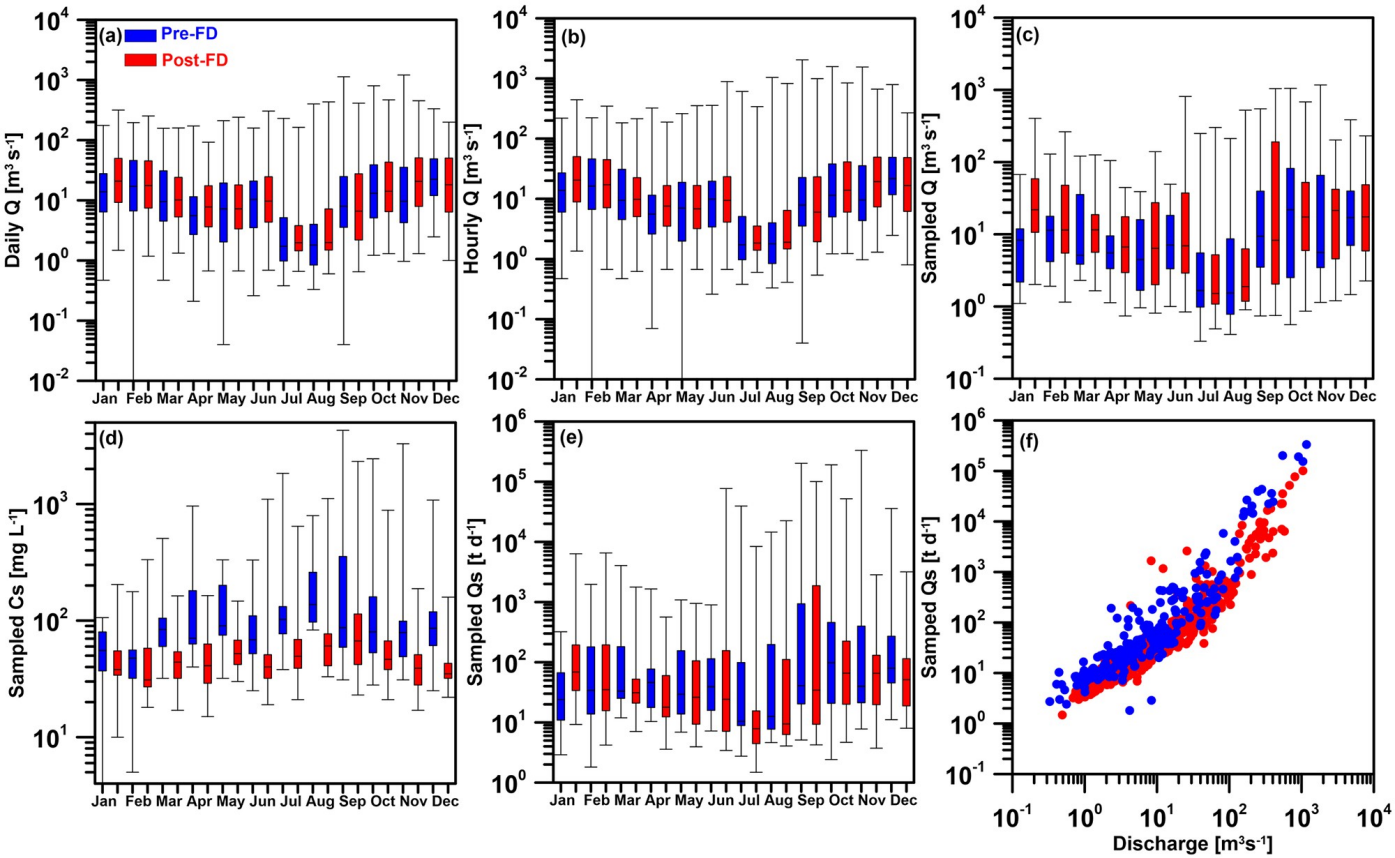
The box and whisker plot of (a) daily discharge, (b) hourly discharge, (c) water discharge at the occasion when sediment samples were taken (sampled *Q*), (d) sampled sediment concentrations (sampled *Cs*), (e) sampled sediment loads (sampled *Qs*) which is the product of (c) and (d), and (f) the scatter plots of sampled sediment loads against sampled *Q* for both before flood diversion (Pre-FD in blue) and after diversion (Post-FD in red) periods.

The impact of the YFDT on the discharge and sediment transport measured at the Wudu gauge station is illustrated in Fig 3. The terms ’Pre-FD’ and ’Post-FD’ represent data before and after the official operation of YFDT, i.e. July 2005. Fig 3a and 3b respectively depict the daily and hourly discharge distributions before and after flood diversion. Regardless of daily or hourly discharge, the distribution ranges of the first quartile and third quartile show relatively similar values before and after flood diversion. However, it does affect the maximum and minimum discharge values for each month. In terms of daily discharge (Fig 3a), the maximum discharge for the months of April, July, September, October, November, and December all experienced a decrease by 80 [m^3^sec^-1^] (from 172 to 92 [m^3^sec^-1^]) in April to 758 [m^3^sec^-1^] (from 1210 to 452 [m^3^sec^-1^]) in November. In terms of the minimum daily discharge, except for December, which decreased by 1.47 [m^3^sec^-1^] (from 2.47 to 1 [m^3^sec^-1^]), the minimum daily discharge for the other months increased, with the increment ranging from 0.08 to 1.17 [m^3^sec^-1^]. As for the hourly discharge (Fig 3b), the changes observed align with those in daily discharge. For the months of April, July, August, September, October, November, and December, the maximum flow all experienced a decrease by 135 [m^3^sec^-1^] (from 323 to 188 [m^3^sec^-1^]) in April to 1047 [m^3^sec^-1^] (from 2040 to 993 [m^3^sec^-1^]) in September. In terms of the minimum hourly discharge, except for December, which decreased, the minimum hourly discharge for the other months increased, with the increment ranging from 0.01to 0.88 m^3^sec^-1^. The reduction in the highest discharge directly demonstrates the effectiveness of the YFDT, which is the primary purpose of YFDT. This is evident from the daily discharge time series chart in Fig 2, where the daily discharge after flood diversion seems to be truncated and rarely exceeds 450 [m^3^sec^-1^]. However, the general increase in the lowest discharge lacks a straightforward explanation. Nevertheless, sediment transport during high flow periods has a decisive impact [13, 29].

Fig 3c–3e respectively illustrate the water discharge at the occasion when sediment was sampled (sampled *Q*), the sampled sediment concentration (sampled *Cs*), and the sampled sediment transport loads (which is the product of the former two terms, converted to [t d^-1^]) for both Pre-FD and Post-FD periods. Fig 3f presents a scatter plot of the sampled sediment transport loads against water discharges. Looking at the median discharge for the sampled *Q* (Fig 3c), the discharge after flood diversion are generally higher for each month (except for June, July, September, and October) than before flood diversion. The highest sampled *Q* after flood diversion are also generally higher for each month (except for April and October to December). This information indicates that, after flood diversion, the WRA tends to collect sediment concentration samples under conditions of higher discharge than before flood diversion.

However, under relatively higher sampled *Q* after flood diversion, the sampled *Cs* for each month is significantly lower after flood diversion. Looking at the median values for each month, in January, it decreased from 55.5 to 38 [mg L^-1^]; in February, from 47.5 to 31 [mg L^-1^]; in March, from 84 to 44 [mg L^-1^]; in April, from 70.5 to 41 [mg L^-1^]; in May, from 90 to 52 [mg L^-1^]; in June, from 68.5 to 40 [mg L^-1^]; in July, from 102.5 to 49.5 [mg L^-1^]; in August, from 137.5 to 60.5 [mg L^-1^]; in September, from 87 to 67 [mg L^-1^]; in October, from 80 to 46.5 [mg L^-1^]; in November, from 79 to 39 [mg L^-1^]; and in December, from 86 to 35 [mg L^-1^]. Despite the increase in sampled *Q* after flood diversion, the decrease in sampled *Cs* results in little difference in sampled *Qs* before and after flood diversion. While the median of sampled *Qs* indicates a decrease for most months (except January, February, and November), the reduction ranges from 2.08 to 31.89 [t d^-1^]. The sampled results also suggest that, at a given water discharge, the sediment transport loads in the river are lower after flood diversion compared to before (Fig 3f). This phenomenon is observed across all the spectrum of discharge, with a more pronounced difference when the discharge exceeds 100 [m^3^sec^-1^].

#### Results of sediment load estimation

**Fig 4:**
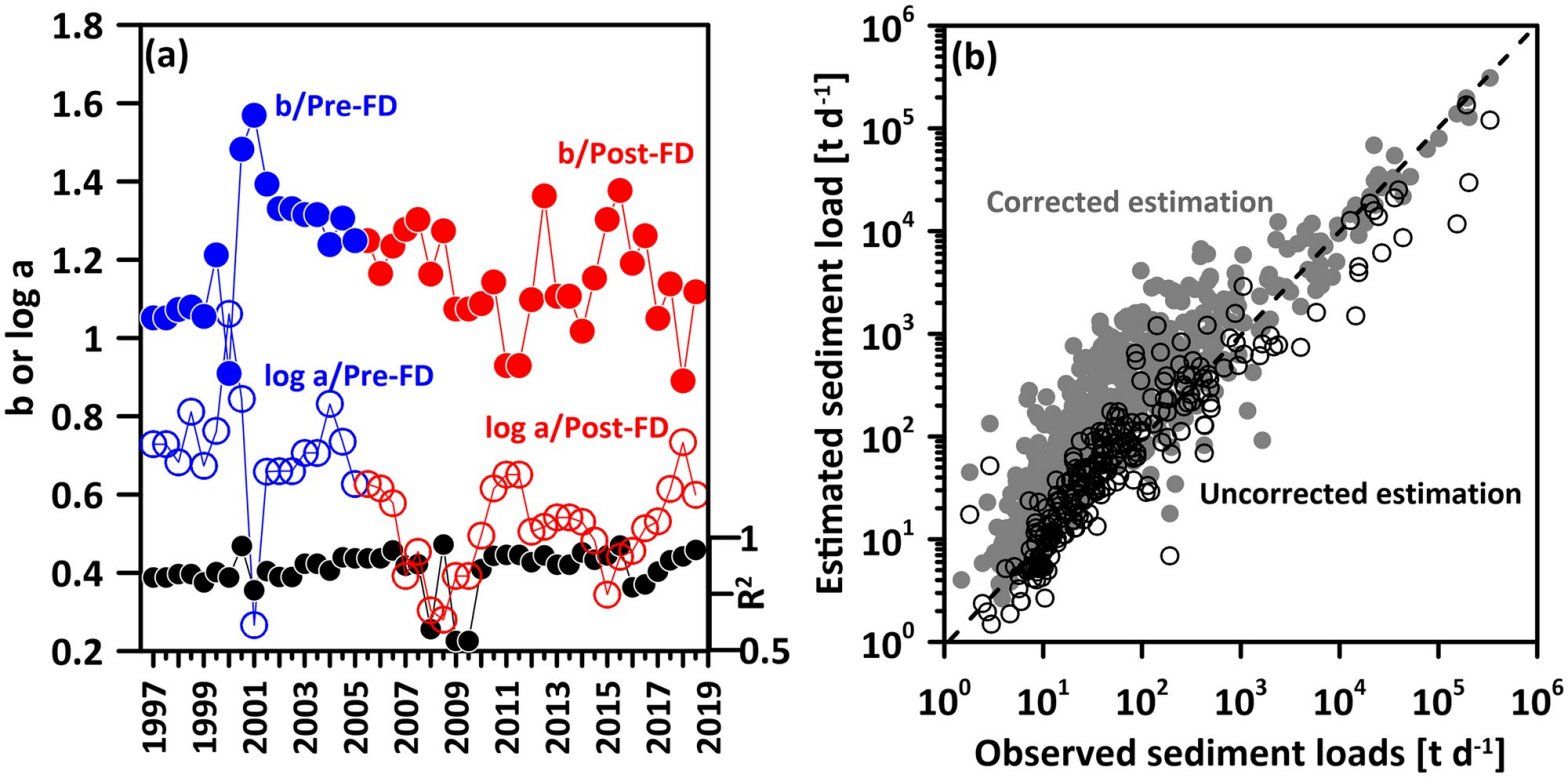
(a) The logarithmic values of the coefficients *a* (open circle), coefficients *b* (colored dot), and the *R*^*2*^ (black dot) of the seasonal rating curves used for sediment load estimation, and (b) the scatter plots of the corrected (grey dot) and uncorrected (open circle) estimation of sediment loads against the observed ones. Blue and red, respectively, indicate before (Pre-FD) and after (Post-FD) the operation of YFDT.

The logarithmic values of the coefficient *a* (hereafter *log a*), exponent *b*, and the *R*^*2*^ of the seasonal rating curves used for sediment load estimation are shown in Fig 4a. Assuming November as the starting month of the hydrological year, each hydrological year corresponds to two rating curves representing sediment transport behaviors with discharge during the low-flow season (November to May) and high-flow season (June to October). Before flood diversion, exponent *b* ranged from 0.91 to 1.57, with an average of 1.23±0.18. After flood diversion, exponent *b* ranged from 0.89 to 1.36, with an average of 1.15±0.13. Before flood diversion, *log a* ranged from 0.27 to 1.06, with an average of 0.71±0.15. After flood diversion, *log a* ranged from 0.28 to 0.73, with an average of 0.51±0.11. Only 3 rating curves had *R*^*2*^ values close to 0.5; the rest were above 0.75, indicating the applicability of using the rating curves for sediment load estimation. Fig 4b illustrates the results of applying the rating curves with and without bias correction to estimate observed sediment loads. After correction, the estimates, especially for the crucial high values in sediment load estimation, generally fall along the 1:1 line. The bias-correction method sacrifices the accuracy of sediment load estimation at lower flow rates to achieve accurate estimation of sediment load at higher flow rates and hence total sediment load during the studies period. When there is a significant underestimation at high values, the overall sediment load will be significantly underestimated. For the upper quantile data, the corrected estimates overestimate the total observed sediment loads by approximately 3%. However, without the correction, the total observed values would be underestimated by 98%. For all the observed data, the corrected estimates overestimate by about 4%, while the uncorrected estimates underestimate by 98%. This also suggests that the estimation for the low-flow data has a lesser impact on the total amount estimation of sediment load.

**Fig 5:**
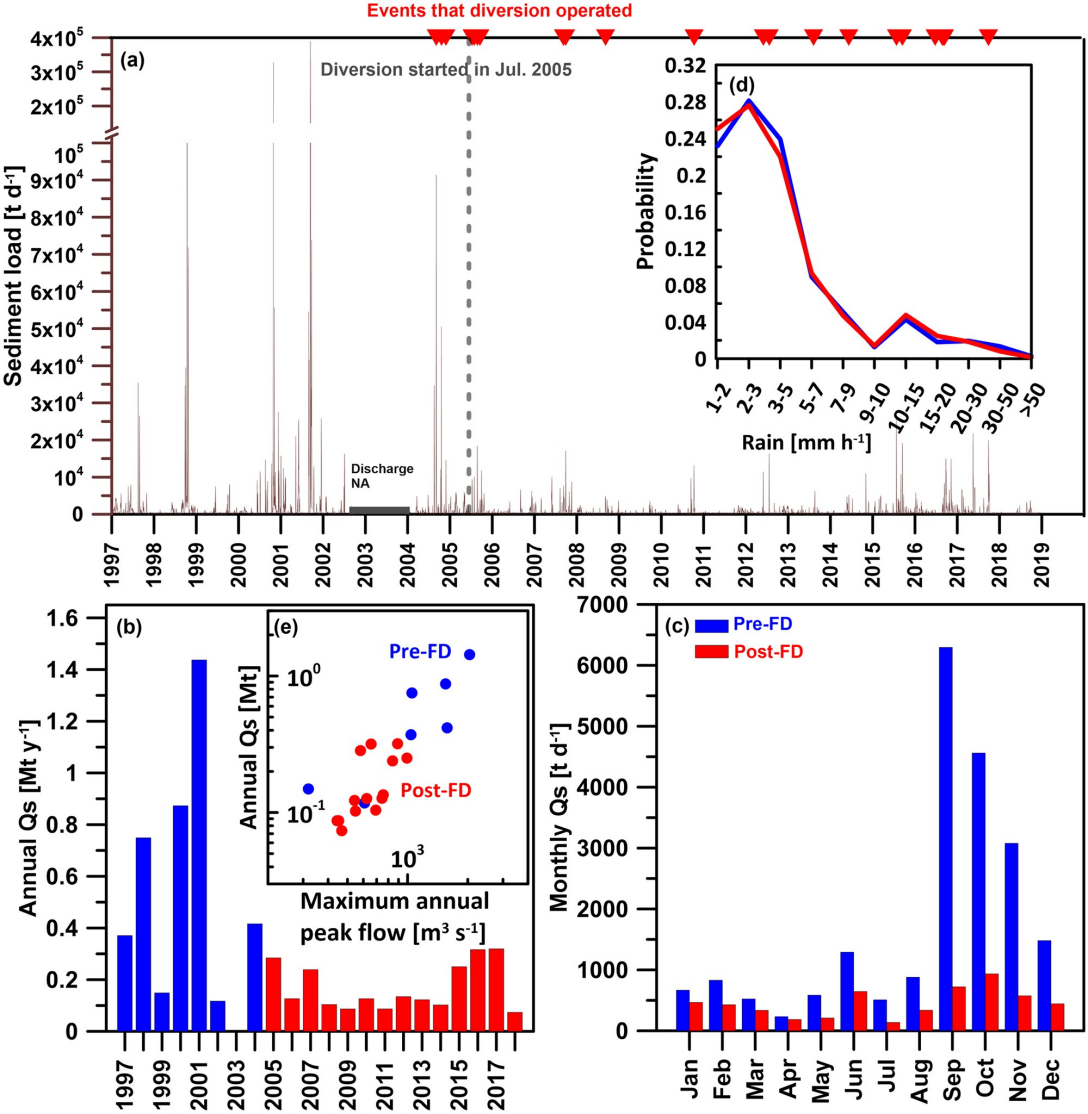
(a) Daily sediment loads, (b) annual sediment loads, (c) monthly averages of sediment loads, (d) probability distribution of hourly rainfall, and (e) relationship between annual sediment loads and maximum annual peak flow before (Pre-FD in blue) and after (Post-FD in red) the operation of the YFDT.

The seasonal rating curves with bias correction factors were then applied to daily (or hourly for the events) discharge to calculate daily sediment loads as shown in Fig 5a. Before flood diversion, the patterns of annual cycles are apparent for sediment loads, peaking in the middle of each year which is mostly associated with episodic events, i.e. typhoons. After flood diversion, the patterns of annual cycles still maintained but the magnitudes of the fluctuations caused by the episodic events significantly dropped. After flood diversion, daily sediment loads hardly reached 30,000 [t d^-1^] which were exceeded almost every year before flood diversion. Before flood diversion, daily sediment loads can exceed 400,000 [t d^-1^] which was >10x higher than the values after flood diversion. Pre-FD and Post-FD daily sediment loads reveal a statistically significant difference according to the Students’ t-test (p-value << 0.01).

The daily sediment loads were then aggregated to obtain annual (Fig 5b) and monthly sediment loads (Fig 5c). Before flood diversion, the total annual sediment loads ranged from 0.12 to 1.44 [Mt y^-1^], with an average of 0.59 ± 0.47 [Mt y^-1^] (Fig 5b). After flood diversion, the total annual sediment loads for each year ranged from 0.074 to 0.32 [Mt y^-1^], with an average of 0.17 ± 0.09 [Mt y^-1^]. There was significant inter-annual variability in sediment loads before flood diversion, while after flood diversion, the yearly sediment loads tended to less fluctuate. Pre-FD and Post-FD yearly sediment loads reveal a statistically significant difference according to the Students’ t-test (p-value < 0.05).

Looking at the monthly averages (Fig 5c), the sediment loads for each month after flood diversion were consistently lower than before flood diversion. Taking the peak period of sediment transport during typhoon season, i.e. August to December, as an example, before flood diversion, the monthly outputs were 880, 6,295, 4,561, 3,079, and 1,482 [t d^-1^], respectively. After flood diversion, the average output for August to December decreased to 338, 723, 935, 576, and 445 [t d^-1^], respectively, with a reduction ranging from 541 to 5,573 [t d^-1^]. In addition to the mentioned months, the average sediment loads for the remaining months also decreased by 46 [t d^-1^] (from 233 to 187 [t d^-1^]) in April to 645 [t d^-1^] (from 1291 to 646 [t d^-1^]) in June. These results indicate a significant reduction in seasonal variation and overall river sediment transport after the operation of YFDT. For most months (except April and November), the daily sediment load within the month for Pre-FD and Post-FD periods shows a statistically significant difference (Student’s t-test, p-value < 0.05). However, the Pre-FD and Post-FD probability distribution of hourly rainfall remained similar (Fig 5d).

## Discussion

### Effects of YFDT on fluvial sediment export

**Fig 6:**
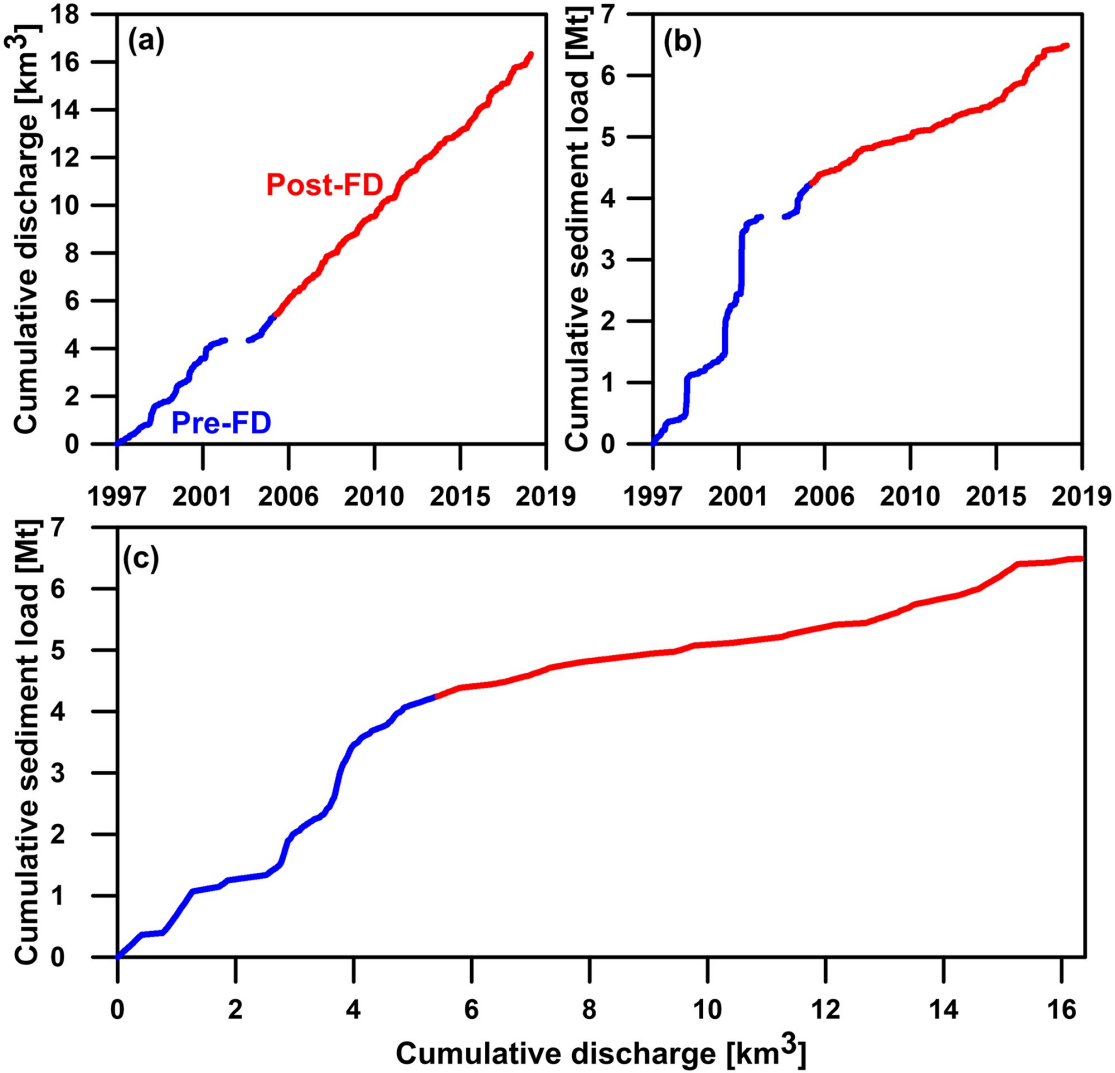
Plots of (a) cumulative curve for daily discharge [km^3^], (b) cumulative curve for daily sediment load [Mt], and (c) double mass curve for cumulative sediment load against cumulative discharge. Curves for before (Pre-FD) and after (Post-FD) the operation of the YFDT are indicated in blue and red, respectively.

The cumulative curves provide insights into the impact of flood diversion on discharge and sediment load. Fig 6a and 6b depict the cumulative curves for daily discharge and sediment load, respectively. The slope of the cumulative discharge curve before flood diversion is approximately 0.0023 [km^3^d^-1^] (Fig 6a), while after flood diversion, the slope is about 0.0022 [km^3^d^-1^]. YFDT’s diversion of peak flows during storm events has a minimal impact on the long-term average discharge. However, it significantly affects sediment load. Before flood diversion, the slope of the cumulative sediment load curve is about 1,538 [t d^-1^] (Fig 6b), and after flood diversion, the slope is approximately 416 [t d^-1^]. Additionally, the Post-FD sediment load cumulative curve is smooth, in contrast to the Pre-FD curve, which shows a stepwise change influenced by the episodic events, i.e. mostly typhoons. Intense typhoons, generally lasting for 3–5 days (~1% of the time in a year), are often responsible for most of the annual sediment loads, which are major features of Taiwanese rivers [13, 31]. The slope of double mass curve further illustrates changes in sediment transport efficiency, i.e., the amount of sediment transported per unit discharge (Fig 6c). Before flood diversion, the sediment transport efficiency is approximately 0.84 [Mt km^-3^], while after flood diversion, it decreases to about 0.19 [Mt km^-3^]. Furthermore, the sediment transport efficiency after flood diversion tends to stabilize, differing from the Pre-FD period where it was prone to sudden increases influenced by flood events.

**Fig 7:**
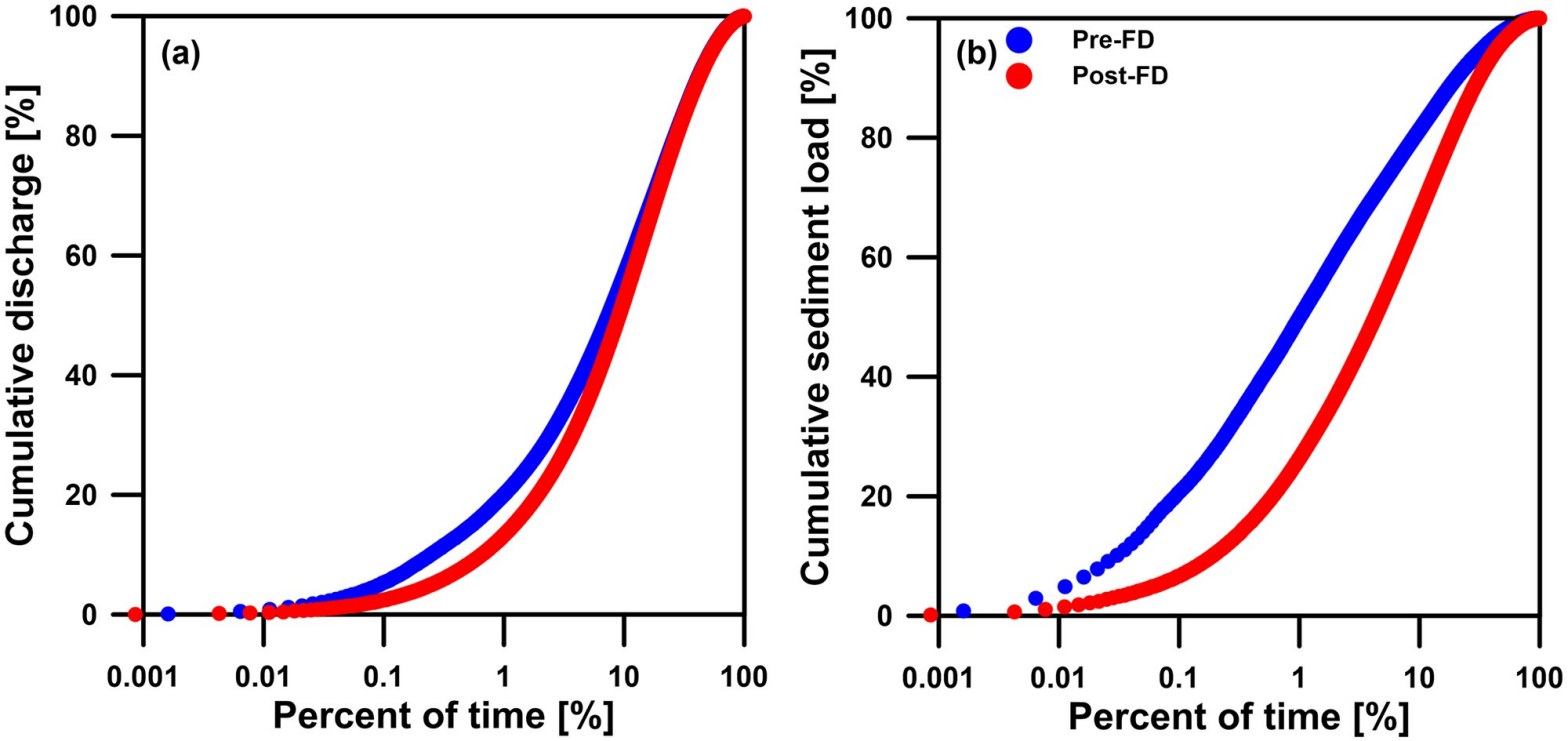
Curves of (a) percent cumulative discharge and (b) percent cumulative sediment load against percent cumulative time over entire record periods of before (Pre-FD in blue) and after (Post-FD in red) the flood diversion.

The drop in the magnitudes of the fluctuations caused by the episodic events indicates a change in the temporal distribution of sediment export, resulting in a more even distribution of sediment export over time. Fig 7b shows the curves of percent cumulative daily sediment loads against percent cumulative time for both the Pre-FD and Post-FD periods, revealing a statistically significant difference (Kolmogorov-Smirnov test, p-value << 0.01). Before flood diversion, the Keelung River requires ~1% cumulative time to export 50% cumulated sediment loads, which is not uncommon in Taiwan. The previous study on 16 major rivers in Taiwan showed that most sediment erosion and delivery occur in response to typhoon-generated floods, as evidenced by the fact that >50% of the long-term sediment export occurs in <1% of time [31]. However, after flood diversion, it takes ~4.5% cumulative time to export 50% cumulated sediment loads. In other words, ~1% cumulative time can merely represent ~25% cumulated sediment loads, indicating sediment load is relatively evenly-exported across time although episodic events still dominate the percent cumulative sediment load. However, the Post-FD sediment delivery efficiency is much lower compared to the Pre-FD condition. Nevertheless, relatively slight changes were found between the Pre-FD and Post-FD curves of precent cumulative daily discharge against percent cumulative time (Fig 7a, showing statistically significant difference after Kolmogorov-Smirnov test with p-value << 0.01), echoing the similar curves of the Pre-FD and Post-FD cumulative discharge in Fig 6a. It takes 1% cumulative time to drain ~20% and ~15% cumulated discharge, respectively, for the Pre-FD and Post-FD periods.

It is evident that the Post-FD annual sediment transport is significantly lower than the Pre-FD one (Fig 5b). Moreover, it is noticeable that the Post-FD sediment transport is closer to the levels observed in 1997, 1999, and 2002. These three years experienced only mild to moderate typhoon invasion on Taiwan, reflecting the downstream Keelung River after flood diversion, where it seems as if there were no severe typhoon invasions. A reduction in peak flow directly impacts sediment load. As shown in the Fig 5e, there is a strong positive correlation between annual sediment load and the maximum annual peak flow, indicating the significant contribution of peak flow to the total annual sediment load. After the flood diversion, the maximum peak flow significantly decreased, leading to a corresponding reduction in annual sediment load. This finding is entirely consistent with previous studies conducted in Taiwan [13]. The Post-FD inter-annual variation in sediment transport tends to be small, with a standard deviation of only 0.09 [Mt] for annual sediment transport, much lower than the Pre-FD value of 0.47 [Mt]. It is important to note that there were indeed typhoon invasions after flood diversion. Moreover, at the condition of Post-FD rainfall patterns remaining the same (Fig 5d), the significant decrease in sediment transport (Fig 5) might be attributed to construction of YFDT rather than climatic factors.

However, the changes in land use in the watershed between YFDT and Wudu gauge station might also affect the reduction in sediment transport at the Wudu gauge station. During the study period, the Ministry of the Interior announced two phases of land use in 1995 (not shown) and 2005 (as Fig 1 shows). The land use distribution in the upstream watershed of YFDT and the watershed between YFDT and Wudu gauge is shown in the Table 1 below. The percentage of various land uses in the upstream watershed of YFDT changed little between the two periods. As for the watershed between YFDT and Wudu gauge, the decrease in grass area in 2005, compared to 1995, is the only change that could potentially lead to a reduction in soil erosion. However, we have some concerns regarding the classification of land use in this area. Based on satellite imagery observations, most of the land classified as grass is actually forest. Even if 3.6% out of the 5.8% classified as grass in 1995 is reclassified as forest (or even if this is not done), we believe that the changes in land use between 1995 and 2005 would not significantly impact the overall sediment load. Since there has been no significant change in rainfall patterns after the flood diversion, we still maintain that the current reduction in sediment load at the Wudu gauge station is mainly due to the flood diversion by the YFDT rather than climatic factors or land use changes. However, this study cannot entirely attribute the reduction in downstream sediment load to the construction of the YFDT alone. Changes in climatic conditions and land use in the downstream and upstream areas over time, although appearing relatively minor, also need to be considered. Better experimental designs and modeling assistance are required to more accurately quantify the contributions of climate, land use, and flood diversion to sediment load changes.

**Table 1:** The percentage distribution of land use in the upstream watershed of YFDT and the watershed between YFDT and Wudu gauge station for the years 1995 and 2005.

Watershed	YFDT-Wudu		Upstream YFDT	
Year	1995	2005	1995	2005
Land use percentage [%]				
Forest	71.4	75.6	91.8	92.6
Agriculture	2.9	3.5	2.5	1.6
Building	15.3	15.9	2.1	3.2
Water	3.7	2.7	1.1	1.5
Grass	5.8	2.2	1.6	1.0
Bare land	0.1	0	0.1	0.1
Other	0.9	0.2	0.9	0

### Preliminary survey of the effects of YFDT on the river continuum

Fluvial sediment export is tightly related to the evolution of river morphology [45–47], the health of downstream habitats and organisms [48], and near-shore aquatic environment [49]. However, most of the previous studies assessing the effects of artificial structures on fluvial sediment export are associated with dams or reservoirs [15]; none of them are associated with flood diversion tunnel. Syvitski et al. (2005) reported differences in sediment load before and after the construction of reservoirs for 217 rivers worldwide [18]. When rivers are classified by landmass, the sediment retained in reservoirs ranged from 0–31%, indicating that downstream sediment load is 69–100% of the pre-reservoir levels. When classified by ocean basin, downstream sediment load was 70–95% of pre-reservoir levels, which is significantly higher than the 29% observed for the YFDT. This suggests that the impact of flood diversion tunnel on downstream sediment load may be greater than that of reservoirs. For another example of the Zengwen Reservoir in southern Taiwan [31], sediment load in the outlet of Zengwen River before reservoir construction was approximately 13 Mt yr^-1^. During the initial 10 years of reservoir impoundment, sediment load decreased to 4 Mt yr^-1^ (about 30% of the original amount), but then slightly recovered to 7 Mt yr^-1^ (about 54% of the original amount). It is also worth noting that sediment trapped by reservoirs is at least retained within the watershed and can be reintroduced downstream through sediment release mechanisms. Whereas the sediment carried away by the flood diversion tunnel will permanently exit the watershed, making its impact irreversible. The long-term impact on sediment budget in the watershed and morphological evolution in the channel may vary significantly. Besides, fluvial sediment load is a good surrogate to infer erosion of mountain belts controlling their topographic and structural evolution [4]. The impact assessment of changes in the sediment load to the coastal zone has become difficult because of the conflicting impacts of humans, e.g. accelerated soil erosion versus reduced sediment load due to water diversion [15]. The observed sediment loads in the downstream of flood diversion tunnels would definitely bias erosion of the watershed. While the YFDT has significantly reduced the risk of downstream flooding in the Keelung River, the chain reaction resulting from the drastic reduction in sediment transport is still worthy of ongoing attention.

To gain a preliminary understanding of the potential impact of the YFDT on water quality, monthly BOD data have been collected from two water quality monitoring stations located upstream (Jieshou station) and downstream (Liuho station) of the YFDT, provided by the Environmental Protection Administration (EPA) from 1994 to 2019. These data were used to assess the impact of YFDT on the export of organic matter in the river. The BOD concentration at the Jieshou station during the Pre-FD period was 1.91±1.22 ppm, while during the Post-FD period, it was 1.80±1.46 ppm, showing no significant difference according to the Students’ t-test. However, there was a significant difference in the Liuho station before and after the flood diversion. The BOD concentration at the Liuho station of YFDT during the Pre-FD period was 4.62±5.76 ppm, and during the Post-FD period, it was 3.24±2.56 ppm (significantly lower by t-test with p-value <<0.05). Despite this, the BOD concentration at the downstream station was consistently higher than that at the upstream station, both before and after the flood diversion. Previous studies analyzing water quality data of the Keelung River from 2002 to 2013 have shown that the dissolved inorganic nitrogen concentration from upstream to downstream in the Keelung River is highly positively correlated with the degree of urbanization, indicating that human activities within urban areas control the variation in downstream water quality [50, 51]. This is also reflected in the significantly higher BOD concentrations downstream compared to upstream. Additionally, due to improvements in sewage treatment facilities, the water quality of the Keelung River has gradually improved even before the operation of the YFDT [51], making it even more challenging to ascertain the impact of the flood diversion on water quality. Besides, the main impacts of the flood diversion occur during typhoon events, which are not covered by the EPA’s water quality data. Therefore, more targeted research designs are still needed in the future.

Regarding the potential ecological impacts, the WRA conducted surveys on fish species and their abundance in the Keelung River in 2005 and 2017 [52]. To assess the ecological impact of the YFDT, we selected one survey section each from upstream (Jieshou (Hodon) station) and downstream (Shuiwenwan station) of YFDT. The survey recorded 33 species of fish from 16 families. Due to the different survey months between the two periods, the comparison is based on the results from the same months. In the upstream survey section of YFDT, there were 7 fish species with a total of 28 individuals recorded in August 2005, and 10 fish species with a total of 91 individuals recorded in August 2017. In the downstream survey section of YFDT, there were 4 fish species with a total of 75 individuals recorded in August and October 2005, and 18 fish species with a total of 196 individuals recorded in August and October 2017. The variability of discharge data, including magnitude, duration, amplitude, frequency, and timing, are linked with their influence on aquatic species across different life stages [53]. The construction and operation of water conservancy projects attenuate such fluctuations and can even disrupt flow, leading to a reduction in the diversity and richness of aquatic organisms [54, 55]. During Post-FD period, less fish species and abundance were not revealed even if the significant decrease in high flow rates (Fig 2) and marked change in the flow distribution (Fig 7a). Therefore, the increase in fish species and abundance in the downstream river section may be more closely related to the improvement in river water quality (mentioned above). Besides, there is also evidence of ecological improvement in the upstream river section where flow regime has not been affected by the YFDT.

### Shifts of sediment transport regime

**Fig 8:**
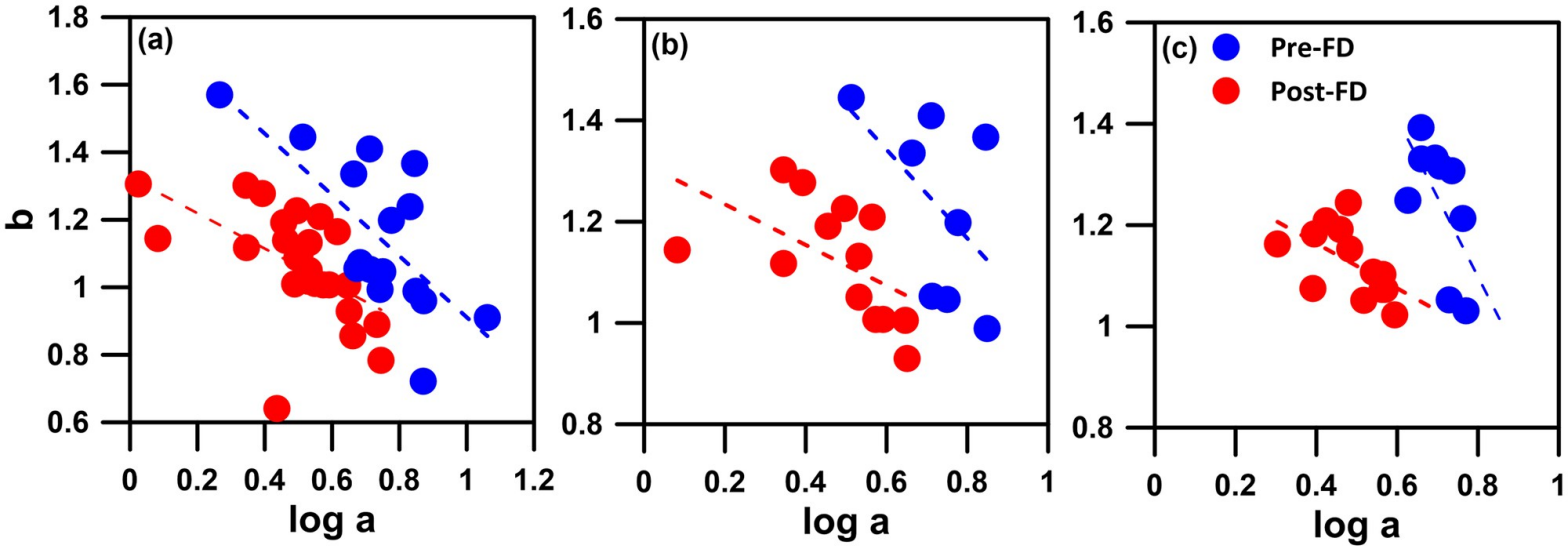
Relationship between *b* and *log a* for both Pre-FD (blue) and Post-FD (red) rating curves developed at (a) seasonal, (b) annual, and (c) three-year scales. The *a* and *b* are the coefficients in the rating curves.

In this study, the relationship between exponent *b* and *log a* representing the sediment transport regime, for both Pre-FD and Post-FD periods, is characterized by different straight lines, irrespective of whether the rating curves are developed at seasonal (Fig 8a), annual (Fig 8b), or three-year scales (Fig 8c). This indicates a change in sediment transport regime before and after the diversion, suggesting that the Post-FD sediment transport regime is akin to that of a different river. Higher position of *b-log a* pairs on the plot correspond to a larger portion of the annual sediment load being transported during high discharge [32]. Both Post-FD *log a* and exponent *b* are smaller than the Pre-FD (Fig 4a), and the straight line representing the Post-FD sediment transport regime is positioned lower in the *b-log a* pairs plot, indicating a smaller portion of the annual sediment load being transported during high discharge. This aligns with the findings presented in Fig 7b.

High *log a* indicate intensively weathered materials, which can easily be transported in the riverbed. The *log a* controls sediment load during low-flow conditions. The exponent *b* represents the erosive power of the river, gradually taking place to control sediment load with increase in discharge. Large *b* value is indicative for rivers where a small increase in discharge results in a strong increase in erosive power of the river. Others state that the exponent *b* indicates the extent to which new sediment sources become available when discharge increases [32]. The lower Post-FD *log a* values indicate a reduction in the sediment that is more easily flushed off in the riverbed, resulting in lower sediment concentration measurements (Fig 3d). A reasonable inference is that this reduction is a consequence of the diversion significantly decreasing the sediment amount transported from the upstream to the downstream. Consequently, there is a decrease in the overall sediment deposition in the riverbed, leading to a significant reduction in sediment transports during low-flow condition when YFDT is not executed (i.e. January to May in Fig 5c). The decrease in exponent *b* suggests a reduction in erosive power, likely associated with the inability to provide additional sediment from the upstream for transport with increase in discharge after diversion.

The variation in coefficients is also linked to the morphology of the river channel. V-shaped valleys, characterized by a smaller contact area between river water and the riverbed during low flow, i.e. smaller wetted perimeter, typically correspond to smaller *log a* values. In contrast, U-shaped valleys, with a larger wetted perimeter, correspond to larger *log a* values. Yang et al. (2007) demonstrated this trend using data from ten gauges along the Yangtze River from 1950 to 1988, showing an increasing trend in *log a* and a transition from V-shaped to U-shaped valleys from upstream to downstream [34]. In this study, the decrease in Post-FD *log a* raises the question of whether it reflects a reduction in sediment supply from the upstream, leading to river incision and a gradual transition toward a more V-shaped morphology [45]. Verification of this hypothesis awaits further on-site investigations. However, in Yang et al.’s (2007) study on the Yangtze River, where the upstream and downstream gauges exhibited the same sediment transport regime, a decrease in *log a* corresponds to an increase in exponent *b* [34]. Considering the river channel morphology, the increase in wetted perimeter during the process of increasing discharge in V-shaped valleys is larger than that in U-shaped valleys. This suggests a higher erosive power in V-shaped valleys, resulting in a larger *b* compared to U-shaped valleys. However, in our study, both Post-FD *log a* and exponent *b* decrease, and Pre-FD and Post-FD sediment transport regimes are not the same. Sun et al. (2020) found the reduced soil erosion led to decrease in *log a* and increase in exponent *b* but different sediment transport regime after the large-scale soil and water conservation measures (called ecological restoration) in the Middle Yellow River Basin [36]. However, they found sediment transport may be higher under extreme discharge despite the ecological restoration, which would never happen in our case.

### Shifts of people’s disaster perception

Although understanding the impact of the YFDT on downstream people’s disaster perception is beyond the scope of this study, we attempted to broaden the impact of our research by addressing this issue. We collected results from related studies from the government-funded project report to understand changes in public disaster perception. Wu (2016) used in-depth interviews and surveys to find that the public has a high level of trust and reliance on the YFDT, with 48% of surveyed communities and households believing that the project can fully mitigate climate change impacts [56]. This contrasts with pre-YFDT survey results [57, 58], where more than half of the respondents held a pessimistic view regarding future flooding conditions. The change in public disaster perception is also reflected in property prices in the Xizhi area (downstream area adjacent to the YFDT), which increased from approximately US$750 per m^2^ before the YFDT construction to approximately NT$2,907 per m^2^ in 2016. This suggests that the construction of YFDT has influenced the disaster perception of downstream communities and households, providing residents with a sense of safety and trust. Wu (2016) further stated that this result echoes the concept of the levee effect described in the literature [56, 59–63], indicating that such engineering projects can lead to a false sense of risk perception among stakeholders. Future research should consider how to mitigate and avoid such phenomena.

## Conclusions

In an effort to mitigate flood discharges within the Keelung River, the Yuanshantze Flood Diversion Tunnel Project was initiated in its upper reaches. This strategic intervention diverted peak flows resulting from typhoon-induced rainfall towards the East China Sea, effectively alleviating downstream flooding issues. However, the diversion’s impact on sediment transport within the river is notably more substantial than its influence on flow, to the extent that it induces a transformation in the sediment transport regime. In other words, the Keelung River is no longer the Keelung River it used to be. The sediment supply originating from the upstream exerts a profound influence on the morphology, riverbed substrate, and coastal alterations downstream. This dynamic significantly impacts the aquatic habitat, while the organic content within the sediment further shapes the nutrient and energy dynamics in the downstream river. The diversion project disrupts the longstanding equilibrium of the river, entering into a novel state of normality. The downstream river’s responses to this new normality necessitate further investigation, including aspects such as the exposure of bridge pier foundations, the fluctuations in mangroves at the estuary, and even the shift of people’s disaster perception.

Previous research has predominantly concentrated on the effects of artificial structures on flow magnitude, with a predominant focus on dams. To our knowledge, none of studies regarding the impacts of flood diversion on sediment transport have been documented. Moreover, the diversion tunnel, by carrying sediment away from the watershed, reveals its differential influence on watershed sediment budget compared to dams. Furthermore, this study emphasizes that, for rivers with high sediment yields, the impact of artificial structures on sediment transport should not be overlooked. The impact generated could be more substantial than those related to flow, and their subsequent consequences may extend more widely than the extent of flow alone. In conclusion, while the diversion tunnel addresses immediate human concerns, whether it triggers a series of cascading effects on topography and ecology remains ongoing attention, including the unprecedented influx of a substantial volume of sediment into the East China Sea.

## Supporting information

S1 Table (A). Water discharge at the occasion when sediment samples were taken (sampled Q [m^3^sec^-1^]), sampled sediment concentrations (sampled Cs [mg L^-1^]), and sampled sediment loads (sampled Qs [t d^-1^]). (B). Historical daily water discharge [m^3^sec^-1^]. (C). Historical hourly water discharge [m^3^sec^-1^].
(XLSX)

## References

[1] Milliman J D and Meade R H. World-wide delivery of river sediment to the oceans. The Journal of Geology. 1983; 91: 1–21.

[2] Syvitski J P, Peckham S D, Hilberman R, & Mulder T. Predicting the terrestrial flux of sediment to the global ocean: a planetary perspective. Sedimentary. Geology. 2003; 162.1–2: 5–24.

[3] Schmidt J C, Parnell R A, Grams P E, Hazel J. E, Kaplinski M A, Stevens L E, et al. The 1996 controlled flood in Grand Canyon: flow, sediment transport, and geomorphic change. Ecological Applications. 2001; 11.3: 657–671.

[4] Dadson S J, Hovius N, Chen H, Dade W B, Hsieh M L, Willett S D, et al. Links between erosion, runoff variability and seismicity in the Taiwan orogen. Nature. 2003; 426.6967: 648–651. doi: 10.1038/nature02150 14668860

[5] Moussa T B, Amrouni O, Hzami A, Dezileau L, Mahe G, Abdeljaouad S. Progradation and retrogradation of the Medjerda delta during the 20th century (Tunisia, Western Mediterranean). Comptes Rendus. Géoscience. 2019; 351.4: 340–350.

[6] Hzami A, Heggy E, Amrouni O, Mahé G, Maanan M, Abdeljaouad S. Alarming coastal vulnerability of the deltaic and sandy beaches of North Africa. Scientific reports. 2021; 11(1): 2320. doi: 10.1038/s41598-020-77926-x 33504845 PMC7840745

[7] Neil D T, Orpin A R, Ridd P V, Yu B. Sediment yield and impacts from river catchments to the Great Barrier Reef lagoon. Marine and Freshwater Research. 2007; 53: 733–752.

[8] Rabalais N N, Turner R E, Gupta B K S, Platon E, Parsons M L. Sediments tell the history of eutrophication and hypoxia in the Northern Gulf of Mexico. Ecological Applications. 2007; 17: S129–S143.

[9] Von Blanckenburg F. The control mechanisms of erosion and weathering at basin scale from cosmogenic nuclides in river sediment. Earth and Planetary Science Letters. 2006; 237: 462–479.

[10] Gabet V, Miège C, Bados P and Coquery M. Analysis of estrogens in environmental matrices. TrAC Trends in Analytical Chemistry. 2007; 26: 1113–1131.

[11] Walling D E. Human impact on land–ocean sediment transfer by the world’s rivers. Geomorphology. 2006; 79: 192–216.

[12] Bao H, Lee T Y, Huang J C, Feng X, Dai M, Kao S J. Importance of Oceanian small mountainous rivers (SMRs) in global land-to-ocean output of lignin and modern biospheric carbon. Scientific Reports. 2015; 5: 16217. doi: 10.1038/srep16217 26584586 PMC4653641

[13] Lee T Y, Huang J C, Lee J Y, Jien S H, Zehetner F, Kao S J. Magnified sediment export of small mountainous rivers in Taiwan: chain reactions from increased rainfall intensity under global warming. PloS One. 2015; 10.9: e0138283. doi: 10.1371/journal.pone.0138283 26372356 PMC4570790

[14] Milliman J D, Farnsworth K L, Jones P D, Xu K H, Smith L C. Climatic and anthropogenic factors affecting river discharge to the global ocean, 1951–2000. Global and planetary change. 2008; 62: 187–194.

[15] Syvitski J P. Supply and flux of sediment along hydrological pathways: research for the 21st century. Global and Planetary Change. 2003; 39: 1–11.

[16] Walling D E, Fang D. Recent trends in the suspended sediment loads of the world’s rivers. Global and Planetary Change. 2003; 39: 111–126.

[17] Syvitski J P, Milliman J D. Geology, geography, and humans battle for dominance over the delivery of fluvial sediment to the coastal ocean. The Journal of Geology. 2007; 115: 1–19.

[18] Syvitski J P, Vörösmarty C J, Kettner A J, Green P. Impact of humans on the flux of terrestrial sediment to the global coastal ocean. Science. 2005; 308: 376–80. doi: 10.1126/science.1109454 15831750

[19] Yang S L, Milliman J D, Li P, Xu K. 50,000 dams later: Erosion of the Yangtze River and its delta. Global and Planetary Change. 2011; 75: 14–20.

[20] Serra-Llobet A, Kondolf G M, Magdaleno F, Keenan-Jones D. Flood diversions and bypasses: Benefits and challenges. Wiley Interdisciplinary Reviews: Water. 2022; 9: e1562.

[21] Chen W B, Liu W C, Fu H S, Jang J H. Assessing the influences of a flood diversion project on mitigating river stage, inundation extent and economic loss. Water. 2015; 7: 1731–1750.

[22] Legates D R. Global and terrestrial precipitation: A comparative assessment of existing climatologies. International Journal of Climatology. 1995; 15: 237–258.

[23] Hwang C. Suspended sediments of Taiwan rivers and their geomorphological significance. Bulletin of Nation Taiwan Normal University. 1982; 27: 649–677.

[24] Milliman J D, Syvitski J P. Geomorphic/tectonic control of sediment discharge to the ocean: the importance of small mountainous rivers. The Journal of Geology. 1992; 100: 525–544.

[25] Mulder T and Syvitski J P 1995 Turbidity currents generated at river mouths during exceptional discharges to the world oceans The Journal of Geology 103 285–299.

[26] Farnsworth K L and Milliman J D 2003 Effects of climatic and anthropogenic change on small mountainous rivers: the Salinas River example Global and Planetary Change 39 53–64.

[27] Liu S C, Fu C, Shiu C J, Chen J P, Wu F. Temperature dependence of global precipitation extremes. Geophysical Research Letters. 2009; 36: 17.

[28] Huang J C, Lee T Y and Lee J Y. Observed magnified runoff response to rainfall intensification under global warming. Environmental Research Letters. 2014; 9: 034008.

[29] Kao S J, Lee T Y, Milliman J D. Calculating highly fluctuated suspended sediment fluxes from mountainous rivers in Taiwan. Terrestrial Atmospheric and Oceanic Sciences. 2005; 16: 653.

[30] Milliman J D, Kao S J. Hyperpycnal discharge of fluvial sediment to the ocean: impact of super-typhoon Herb on Taiwanese rivers. Journal of Geology. 2005; 113: 503–516.

[31] Kao S J, Milliman J D. Water and sediment discharge from small mountainous rivers, Taiwan: the roles of lithology, episodic events, and human activities The Journal of Geology. 2008; 116: 431–448.

[32] Asselman N E M. Fitting and interpretation of sediment rating curves. Journal of Hydrology. 2000; 234: 228–248.

[33] Syvitski J P, Morehead M D, Bahr D B, Mulder T. Estimating fluvial sediment transport: the rating parameters Water Resources Research. 2000; 36: 2747–2760.

[34] Yang G, Chen Z, Yu F, Wang Z, Zhao Y, Wang Z. Sediment rating parameters and their implications: Yangtze River, China. Geomorphology. 2007; 85(3–4): 166–175.

[35] Fan X, Shi C, Zhou Y, Shao W. Sediment rating curves in the Ningxia-Inner Mongolia reaches of the upper Yellow River and their implications Quaternary International. 2012; 282: 152–162.

[36] Sun P, Wu Y, Gao J, Yao Y, Zhao F, Lei X, et al. Shifts of sediment transport regime caused by ecological restoration in the Middle Yellow River Basin. Science of the Total Environment. 2020; 698: 134261. doi: 10.1016/j.scitotenv.2019.134261 31783458

[37] Shimizu H, Hamada H, Yasunaga T, Taira K and Kuniya M. Planning and construction of Keelung River Yuanshantzu Flood-Diversion Tunnel Project. 1st ed. London: CRC Press; 2005.

[38] Ferguson R. I. River loads underestimated by rating curves. Water resources research. 1986; 22(1): 74–76.

[39] Salarijazi M, Abdolhosseini M, Ghorbani K, Eslamian S. Evaluation of quasi-maximum likelihood and smearing estimator to improve sediment rating curve estimation. International Journal of Hydrology Science and Technology. 2016; 6.4: 359–370.

[40] Thomas R B. Estimating total suspended sediment yield with probability sampling. Water Resources Research. 1985; 21(9): 1381–1388.

[41] Cohn T A, Delong L L, Gilroy E J, Hirsch R M, Wells D K. Estimating constituent loads. Water resources research. 1989; 25.5: 937–942.

[42] Duan N. Smearing estimate: a non-parametric retransformation method. Journal of the American Statistical Association. 1983; 78: 605–610.

[43] Ghadim H B, Salarijazi M, Ahmadianfar I, Heydari M, Zhang T. Developing a Sediment Rating Curve Model Using the Curve Slope. Polish Journal of Environmental Studies. 2020; 29.2: 1151–1159.

[44] Salarijazi M, Modabber-Azizi S, Mohammadi M, Mohammadrezapour O, Ghorbani K. Development of Suspended Sediment Rating Curve Model by Statistical Classification of River Discharge Data (Case Study: Ghareh-Sou Coastal Watershed). Iranian Journal of Science and Technology, Transactions of Civil Engineering. 2024, 1–10.

[45] Williams G P, Wolman M G. 1984 Downstream effects of dams on alluvial rivers. Washington: Geological Survey Professional Paper 1286; 1984.

[46] Turowski J M, Hovius N, Meng-Long H, Lague D and Men-Chiang C. Distribution of erosion across bedrock channels Earth Surface Processes and Landforms: The Journal of the British Geomorphological Research Group. 2008; 33: 353–363.

[47] Turowski J M, Badoux A, Leuzinger J and Hegglin R. Large floods, alluvial overprint, and bedrock erosion Earth Surface Processes and Landforms. 2013; 38: 947–958.

[48] Ligon F K, Dietrich W E and Trush W J. Downstream ecological effects of dams: A geomorphic perspective. BioScience. 1995; 45: 183–192.

[49] Dai S B, Yang S L, Cai A M. Impacts of dams on the sediment flux of the Pearl River, southern China. Catena. 2008; 76: 36–43.

[50] Lee T Y, Shih Y T, Huang J C, Kao S J, Shiah F K, Liu K K. Speciation and dynamics of dissolved inorganic nitrogen export in the Danshui River, Taiwan. Biogeosciences. 2014; 11: 5307–5321. doi: 10.5194/bg-11-5307-2014

[51] Kuo N W, Jien S H, Hong N M, Chen Y T, Lee T Y. Contribution of urban runoff in Taipei metropolitan area to dissolved inorganic nitrogen export in the Danshui River, Taiwan. Environmental Science ad Pollution Research. 2017; 24: 578–590. doi: 10.1007/s11356-016-7825-4 27738864

[52] National Taiwan University. Investigation of Current Status in Tamsui River System (3/3). 1 st ed. New Taipei City: Tenth River Management Branch, Water Resource Agency, Ministry of Economic Affairs. 2017.

[53] Richter B D. A Method for Assessing Hydrologic Alteration within Ecosystems Conservation Biology. 1996; 10: 1163–1174.

[54] Reid A J, Carlson A K, Creed I F, Eliason E J, Gell P A, Johnson P T et al. Emerging threats and persistent conservation challenges for freshwater biodiversity. Biological Reviews. 2019; 94(3): 849–873 doi: 10.1111/brv.12480 30467930

[55] Su G H, Logez M, Xu J, Tao S L, Villéger S, Brosse S. Human impacts on global freshwater fish biodiversity. Science. 2021; 371(6531): 835–838. doi: 10.1126/science.abd3369 33602854

[56] Wu J Y. Risk Communication on Disaster Adaptations in the River Basin Areas under Climate Change: the Cases of Households and Communities in the Keelung River Basin II. Ministry of Science and Technology. 2016.

[57] Luo K C. The Residents’ Flood Perceptions and Adjustments: A Case Study in Xi-Zhi, Taipei County. M.Sc. Thesis, National Taiwan University. 2003. https://hdl.handle.net/11296/chkz8w.

[58] Lu K Y. The Study of the Residents’ Hazard Perceptions and Adjustments about the Flood in Sijhih City. M.Sc. Thesis, National Don Hwa University. 2005. https://hdl.handle.net/11296/8352nc.

[59] Birkland T A, Burby R J, Conrad D, Cortner H, Michener W K. River ecology and flood hazard mitigation. Natural Hazards Review. 2003; 4(1): 46–54.

[60] Cutter S L, Emrich C T. Moral hazard, social catastrophe: The changing face of vulnerability along the hurricane coasts. The Annals of the American Academy of Political and Social Science. 2006; 604.1: 102–112.

[61] Burby R. J. Hurricane Katrina and the paradoxes of government disaster policy: Bringing about wise governmental decisions for hazardous areas. The annals of the American academy of political and social science. 2006; 604.1: 171–191.

[62] Anderson W., Kjar S. A. Hurricane Katrina and the levees: taxation, calculation, and the matrix of capital. International Journal of Social Economics. 2008; 35.8: 569–578.

[63] Deegan, M. A. Exploring US Flood Mitigation Policies: A Feedback View of System Behavior. Ph.D Thesis. The State University of New York at Albany. 2007. https://0-www.proquest.com.opac.lib.ntnu.edu.tw/dissertations-theses/exploring-u-s-flood-mitigation-policies-feedback/docview/304747315/se-2?accountid=14228

